# Neurological failure in ICU patients with hematological malignancies: A prospective cohort study

**DOI:** 10.1371/journal.pone.0178824

**Published:** 2017-06-09

**Authors:** Chiara Marzorati, Djamel Mokart, Frederic Pène, Virginie Lemiale, Achille Kouatchet, Julien Mayaux, François Vincent, Martine Nyunga, Fabrice Bruneel, Antoine Rabbat, Christine Lebert, Pierre Perez, Dominique Benoit, Giuseppe Citerio, Elie Azoulay, Stephane Legriel, on behalf of the Groupe de Recherche en Réanimation Respiratoire en Onco-Hématologie (GRRR-OH)

**Affiliations:** 1 School of Medicine and Surgery, Milano Bicocca University, Milan, Italy; 2 Paoli-Calmettes Institute, Medical-Surgical ICU, Marseille, France; 3 Cochin University Hospital, Medical Intensive Care Unit, AP-HP, Paris, France; 4 Saint-Louis University Hospital, Medical Intensive Care Unit, AP-HP, Paris, France; 5 Angers University Hospital, Intensive Care Unit, Angers, France; 6 Pitié-Salpêtrière University Hospital, Respiratory and Critical Care Department, Paris, France; 7 Avicenne University Hospital, Intensive Care Unit, Bobigny, France; 8 Victor Provo Hospital, Intensive Care Unit, Roubaix, France; 9 Centre Hospitalier de Versailles, Medical-Surgical Intensive Care Unit, Le Chesnay, France; 10 Hotel Dieu University Hospital, Intensive Care Unit, Paris, France; 11 Montaigu Hospital, Intensive Care Unit, La Roche-sur-Yon, France; 12 Brabois University Hospital, Intensive Care Unit, Nancy, France; 13 Ghent University Hospital, Intensive Care Unit, Ghent, Belgium; 14 ECSTRA team, Biostatistics and Clinical Epidemiology, UMR 1153 (Center for Epidemiology and Biostatistics, Sorbonne Paris Cité, CRESS), INSERM, Paris Diderot Sorbonne University, Paris, France; Hospital Sirio-Libanes, BRAZIL

## Abstract

**Background:**

Epidemiological studies of neurological complications in patients with hematological malignancies are scant. The objective of the study was to identify determinants of survival in patients with hematological malignancy and neurological failure.

**Methods:**

Post hoc analysis of a prospective study of adults with hematological malignancies admitted for any reason to one of 17 university or university-affiliated participating ICUs in France and Belgium (2010–2012). The primary outcome was vital status at hospital discharge.

**Results:**

Of the 1011 patients enrolled initially, 226 (22.4%) had neurological failure. Presenting manifestations were dominated by drowsiness or stupor (65%), coma (32%), weakness (26%), and seizures (19%). Neuroimaging, lumbar puncture, and electroencephalography were performed in 113 (50%), 73 (32%), and 63 (28%) patients, respectively. A neurosurgical biopsy was done in 1 patient. Hospital mortality was 50%. By multivariate analysis, factors independently associated with higher hospital mortality were poor performance status (odds ratio [OR], 3.99; 95%CI, 1.82–9.39; *P* = 0.0009), non-Hodgkin’s lymphoma (OR, 2.60; 95%CI, 1.35–5.15; *P* = 0.005), shock (OR, 1.95; 95%CI, 1.04–3.72; *P* = 0.04), and respiratory failure (OR, 2.18; 95%CI, 1.14–4.25; *P* = 0.02); and factors independently associated with lower hospital mortality were GCS score on day 1 (OR, 0.88/point; 95%CI, 0.81–0.95; *P* = 0.0009) and autologous stem cell transplantation (OR, 0.25; 95%CI, 0.07–0.75; *P* = 0.02).

**Conclusions:**

In ICU patients with hematological malignancies, neurological failure is common and often fatal. Independent predictors of higher hospital mortality were type of underlying hematological malignancy, poor performance status, hemodynamic and respiratory failures, and severity of consciousness impairment. Knowledge of these risk factors might help to optimize management strategies.

## Introduction

Recent advances in the management of hematological malignancies,[1] including the introduction of new drugs, have improved survival and changed the circumstances of admission to the intensive care unit (ICU). New studies of organ support techniques[2–7] have produced refinements in management strategies.[8, 9]

Neurological manifestations have been reported in up to 17% of patients with hematological malignancies.[10] They cover a broad spectrum, as described in several reviews.[11–13] Neurological involvement may be direct, i.e., due to infiltration of the central nervous system (CNS) by the malignant cells; or indirect, i.e., caused by vascular, metabolic, toxic, infectious, or paraneoplastic events.[11, 13]

Epidemiological studies of neurological complications in patients with hematological malignancies are scant and generally relied on retrospective data collection. Few of them focused on severe complications requiring admission to the ICU,[10] which were found to carry a hospital mortality rate of 45%.[10] Further information would help clinicians to identify those patients most likely to benefit from aggressive diagnostic and therapeutic strategies.

To help fill this knowledge gap, we conducted a prospective cohort study in a large population of patients with hematological malignancies who required ICU admission for any reason. Here, we focus on a post hoc analysis of the incidence, causes, outcomes, and risk factors of neurological complications.

## Patients and methods

The appropriate ethics committees in France (CEERB Bichat, 0235) and Belgium (Ghent University Hospital Ethical committee) approved this noninterventional prospective cohort study. In accordance with French law, the CEERB Bichat ethics committee waived the requirement for obtaining written consent from participants or next of kin. Participants or next of kin were given an informed consent document and all of them gave a verbal consent. This was traced and notified in the hospital chart. In accordance with Belgium law, the Ghent University Hospital Ethical committee required a written consent from all participants. Finally, in both countries, informed consent was obtained from all participants or next of kin included in the study.[14]

### Patients

We included consecutive patients with hematological malignancies who were admitted for any reason to one of 17 ICUs in university or university-affiliated hospitals in France and Belgium between January 1, 2010, and May 1, 2011. Noninclusion criteria were age younger than 18 years, ICU admission only to maximize the safety of a procedure, and malignancy in remission for the past 5 years or longer. ICU patients received follow-up for 1 year after ICU discharge. Neurological failure was defined as a neurological disorder requiring ICU admission for monitoring or organ support. The neurological disorder could consist in consciousness impairment, a seizure with or without status epilepticus, a focal sign, encephalopathy, and/or meningeal symptoms. Coma was defined as a score of ≤8.

### Data collection

**Fig 1:**
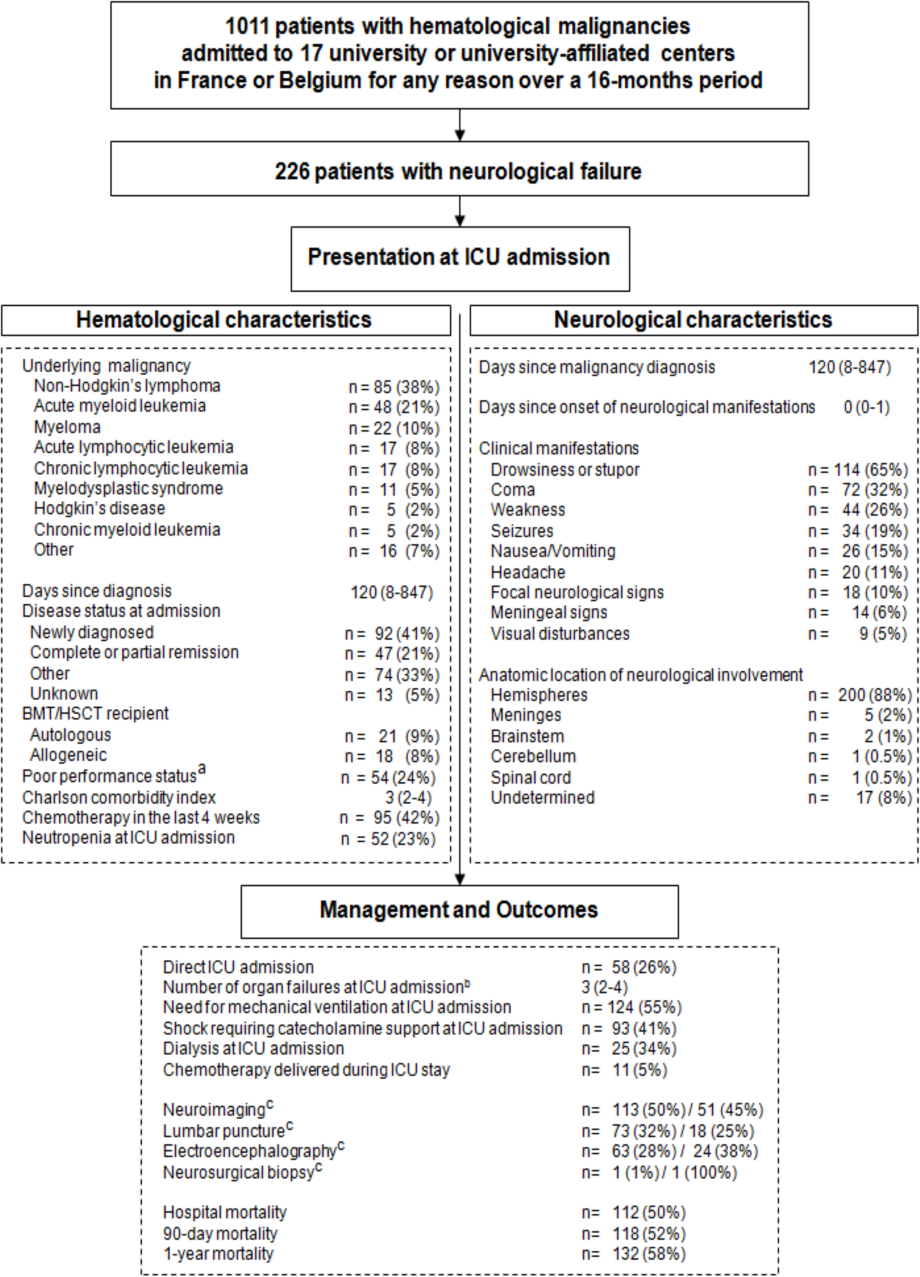
Patient flow chart. ^a^ defined as bedridden or completely disabled. ^b^ number of organ failures at ICU admission defined according to the SOFA score. ^c^ investigations for a cause. First value indicates total number, and second value indicates when the exploration was deemed directly contributive to the final diagnosis.

A standardized electronic form was used to collect the study data reported in Tables 1–3 and Fig 1. Severity and organ dysfunction at ICU admission were assessed using the Simplified Acute Physiology Score II (SAPS-II)[15] and Sepsis-Related Organ Failure Assessment (SOFA) score.[16] The SOFA score was also collected routinely on days 2, 3, 5, and 7 after ICU admission.

**Table 1:** Spectrum of acute organ failures on the first ICU day according to type of hematological malignancy in 1011 patients requiring ICU admission.

	N (%) or Median (Interquartile Range)									
	All patients	Non-Hodgkin's lymphoma	Acute Myeloid Leukemia	Myeloma	Acute Lymphophocytic Leukemia	Chronic Lymphocytic Leukemia	Myelodysplastic Syndrome	Hodgkin's Disease	Chronic Myeloid Leukemia	Other
	n = 1011	n = 320	n = 275	n = 126	n = 76	n = 76	n = 46	n = 25	n = 19	n = 51
**Organ failures on ICU day 1**										
**Neurological**	**226 (22.3%)**	**85 (26.6%)**	**48 (17.5%)**	**22 (17.5%)**	**17 (22.4%)**	**17 (22.4%)**	**11 (23.9%)**	**5(20.0%)**	**5 (26.3%)**	**16 (31.4%)**
Hemodynamic	428 (42.3%)	146 (45.6%)	106 (38.5%)	51 (40.5%)	30 (39.4%)	33 (43.4%)	29 (63.0%)	11 (44.0%)	7 (36.8%)	15 (29.4%)
Respiratory	632 (62.5%)	177 (55.3%)	173 (62.9%)	85 (67.5%)	38 (50.0%)	56 (73.7)	32 (69.6%)	17 (68.0%)	16 (84.2%)	38 (74.5%)
Hematological	194 (19.2%)	52 (16.3%)	76 (27.6%)	17 (13.5%)	10 (13.2%)	13 (17.1%)	11 (23.9%)	5 (20.0%)	4 (21.1%)	6 (11.7%)
Hepatic	83 (8.2%)	29 (9.1%)	26 (9.4%)	8 (6.3%)	2 (2.6%)	1 (1.3%)	4 (8.7%)	3 (12.0%)	3 (15.8%)	7 (13.7%)
Renal	308 (30.5%)	115 (35.9%)	66 (24.0%)	53 (42.1%)	18 (23.7%)	19 (25%)	12 (26.1%)	5 (20.0%)	5 (26.3%)	15 (29.4%)
**Total**	2 (1–2)	2 (1–3)	2 (1–2)	2 (1–2)	1 (1–2)	2 (1–3)	2 (1–3)	1(1–2)	2 (1–3)	2(1–2)

**Table 2:** Neurological features in 226 patients with hematological malignancies and neurological failure requiring ICU admission.

	All patients
	n = 226 (100%)
**General signs**	
Seizures	34 (19.2%)
Nausea and vomiting	26 (14.9%)
**Mental status**	
Headache	20 (11.3%)
Drowsiness or stupor	114 (64.8%)
Coma	72 (32.6%)
**Cranial nerves**	
Any cranial nerve abnormalities	9 (5.1%)
Visual disturbances^a^	9 (5.3%)
**Motor system**	
Weakness	44 (25.9%)
Focal neurological signs^b^	18 (7.9%)
Sphincter tone abnormalities	3 (1.7%)
**Sensory system**	
Any sensory abnormality	4 (2.3%)
Back pain	5 (2.9%)
Radicular pain	2 (1.2%)
Tuft sign	1 (0.6%)
**Cerebellar syndrome**	2 (1.2%)
**Abnormal gait**	34 (19.8%)
**Meningeal signs**	14 (8.0%)
**Brainstem signs**	1 (0.6%)
**Peripheral neurological signs**	2 (1.1%)

^a^Diplopia (n = 3), decreased visual acuity (n = 2), blindness (n = 1), exophthalmia (n = 1), anisocoria (n = 1), mydriasis (n = 1)

^b^Focal neurological signs were defined as symptoms or signs consistent with damage to, or dysfunction of, a specific anatomic site in the central nervous system. Signs were classified as unifocal or multifocal and as transient or persistent; they consisted of hemiparesis (n = 6), hemiplegia (n = 9), monoparesis (n = 2), and paraplegia (n = 1).

**Table 3:** Patient characteristics and predictors of hospital mortality identified by logistic regression.

		N (%) or Median (InterQuartile Range)			Univariate analysis		Multivariable analysis	
		All patients	Survivors	Nonsurvivors	OR (95%CI)	*P* Value	OR (95%CI)	*P* value
		n = 226 (100%)	n = 114 (50.4%)	n = 112 (49.6%)				
**Patient characteristics**								
Age (years), median (IQR)		63 (53–72)	61 (51–72)	64 (54–72)	1.01 (0.99–1.03)	0.21		
Male gender		132 (58.4%)	67 (58.8%)	65 (58.0%)	0.97 (0.57–1.65)	0.91		
Poor performance status^a^		54 (23.9%)	16 (14.0%)	38 (33.9%)	3.15 (1.63–6.07)	**0.0006**	4.44 (2.10–9.93)	**0.0001**
Charlson comorbidity index, median (IQR)		3 (2–4)	3 (2–4)	2 (2–4)	0.78 (0.49–1.23)	0.28		
**Characteristics of the underlying malignancy**								
Classification								
	Non-Hodgkin’s lymphoma	85 (37.6%)	34 (29.8%)	51 (45.5%)	1.97 (1.14–3.40)	**0.01**	2.60 (1.35–5.15)	**0.005**
	Acute myeloid leukemia	48 (21.2%)	24 (21.1)	24 (21.4%)	1.02 (0.54–1.93)	0.94		
	Myeloma	22 (9.7%)	14 (12.3%)	8 (7.1%)	0.55 (0.22–1.37)	0.20		
	Acute lymphocytic leukemia	17 (7.5%)	12 (10.5%)	5 (4.5%)	0.40 (0.14–1.17)	0.09		
	Chronic lymphocytic leukemia	17 (7.5%)	12 (10.5%)	5 (4.5%)	0.40 (0.14–1.17)	0.09		
	Myelodysplastic syndrome	11 (4.9%)	7 (6.1%)	4 (3.6%)	0.57 (0.16–1.99)	0.38		
	Chronic myeloid leukemia	5 (2.2%)	2 (1.7%)	3 (2.7%)	1.54 (0.25–9.40)	0.64		
	Hodgkin’s disease	5 (2.2%)	3 (2.6%)	2 (1.8%)	0.67 (0.11–4.10)	0.67		
	Other	16 (7.1%)	6 (5.3%)	10 (8.9%)	1.76 (0.62–5.03)	0.29		
Newly diagnosed		92 (40.7%)	41 (37.3%)	51 (46.8%)	1.48 (0.86–2.54)	0.15		
Time since hematologic malignancy diagnosis (days), median (IQR)		120 (8–847)	180 (14–1018)	88 (5–482)	1.00 (1.00–1.00)	0.31		
Number of previous chemotherapy lines, median (IQR)		1 (0–2)	1 (0–2)	1 (0–2)	0.96 (0.79–1.16)	0.66		
Allogeneic stem cell transplantation		18 (8.0%)	8 (7.0%)	10 (9.0%)	1.31 (0.50–3.46)	0.58		
Autologous stem cell transplantation		21 (9.3%)	15 (13.2%)	6 (5.4%)	0.38 (0.15–0.97)	**0.04**	0.25 (0.07–0.75)	**0.02**
Remission (complete or partial)		47 (22.1%)	32 (29.6%)	15 (14.3%)	0.40 (0.20–0.79)	**0.008**		
**Circumstances of ICU admission**								
Shock		102 (45.1%)	42 (36.8%)	60 (53.6%)	1.98 (1.16–3.37)	**0.01**	1.95 (1.04–3.72)	**0.04**
Acute respiratory failure		130 (57.5%)	56 (49.1%)	74 (66.1%)	2.02 (1.18–3.45)	**0.01**	2.18 (1.14–4.25)	**0.02**
Acute kidney injury		69 (30.5%)	28 (24.6%)	41 (36.6%)	1.77 (1.00–3.15)	0.05		
GCS score, median (IQR)		12 (7–15)	13 (10–15)	11 (5–14)	0.91 (0.85–0.97)	**0.002**	0.87 (0.81–0.94)	**0.0005**
Neutropenia		52 (23.4%)	24 (21.2%)	28 (26.7%)	1.28 (0.69–2.39)	0.43		
**Severity scores at ICU admission**								
SAPS II^b^, median (IQR)		58 (47–70)	54 (43–60)	64 (53–80)	1.05 (1.02–1.08)	**0.0003**		
SOFA score^b^, median (IQR)		9 (5–13)	7 (4–10)	11 (8–14)	1.20 (1.11–1.30)	**< 0.0001**		
Time to admission since onset of neurological manifestations (days), median (IQR)		0 (0–1)	0 (0–1)	0 (0–1)	1.00 (0.99–1.01)	0.41		
**Cause of neurological manifestations (one or more)**								
	Direct CNS involvement	11 (4.9%)	6 (5.3%)	5 (4.5%)	0.84 (0.25–2.84)	0.78		
	CNS infiltration	11 (4.9%)	6 (5.3%)	5 (4.5%)	0.84 (0.25–2.84)	0.78		
	Indirect CNS involvement	114 (50.4%)	62 (54.4%)	52 (46.4%)	0.73 (0.43–1.23)	0.23		
	Metabolic	73 (32.3%)	39 (34.2%)	34 (30.4%)	0.84 (0.48–1.47)	0.54		
	Iatrogenic	15 (6.6%)	11 (9.6%)	4 (3.6%)	0.35 (0.11–1.12)	0.08		
	Vascular damage	14 (6.2%)	7 (6.1%)	7 (6.3%)	1.02 (0.35–3.01)	0.97		
	CNS infection	12 (5.3%)	5 (4.4%)	7 (6.3%)	1.45 (0.45–4.72)	0.53		
	Nonneurological cause	77 (34.1%)	35 (30.7%)	42 (37.5%)	1.35 (0.78–2.35)	0.28		
	Shock	52 (23.0%)	23 (20.2%)	29 (25.9%)	1.38 (0.74–2.58)	0.31		
	Cardiac arrest	15 (6.6%)	4 (3.5%)	11 (9.8%)	3.00 (0.92–9.71)	0.07		
	Sepsis	10 (4.4%)	8 (7.0%)	2 (1.8%)	0.24 (0.05–1.16)	0.07		
	Undetermined	24 (10.6%)	11 (9.6%)	13 (11.6%)	1.23 (0.53–2.87)	0.63		
**Treatment in the ICU**								
Catecholamine support		132 (58.4%)	45 (39.5%)	87 (77.7%)	5.34 (2.98–9.55)	**<0.0001**		
Noninvasive ventilation		46 (20.4%)	20 (17.5%)	26 (23.2%)	1.42 (0.74–2.73)	0.29		
Mechanical ventilation		154 (68.1%)	59 (51.7%)	95 (84.8%)	5.21 (2.76–9.82)	**<0.0001**		
Dialysis		62 (27.4%)	21 (18.4%)	41 (36.6%)	2.56 (1.39–4.71)	**0.003**		
Emergent anticancer chemotherapy		11 (4.9%)	7 (6.1%)	4 (3.6%)	0.57 (0.16–1.99)	0.38		

Data are number (%) unless otherwise specified. ICU, intensive care unit; SAPS II, Simplified Acute Physiology Score; SOFA score, Sequential Organ Failure Assessment score; OR, odds ratio; 95%CI, 95% confidence interval. Goodness of fit (Le Cessie-Van Houweligen) chi-square *P* = 0.94; the area under the ROC curve estimated by the c statistic was 0.77; *P* values indicating a significant difference (<0.05) are in bold type.

^a^bedridden/completely disabled

^b^Higher scores indicate greater severity.

Malignancies diagnosed within the past 4 weeks were defined as newly diagnosed. The performance status[5] and Charlson comorbidity index[17] were determined at ICU admission. Neutropenia was defined as a neutrophil count less than 500/mm^3^.[18]

Patient management and ICU-admission criteria remained unchanged throughout the study period. A thorough neurological evaluation including determination of the GCS score[19, 20] was performed at ICU admission then on days 2, 3, 5, and 7.

### Management

Mechanical ventilation was used according to standardized criteria in patients whose GCS score remained below 8 despite etiological treatment when a reversible cause was suspected (e.g., status epilepticus or opioid or benzodiazepine poisoning). On-scene intubation was required in some of the comatose patients. Finally, intubation was also required in some of the patients with aspiration pneumonia, respiratory failure, shock, or cardiac arrest.

Vasoactive drugs were restricted to patients with hypotension despite fluid resuscitation or with heart failure documented by echocardiography and evidence of another organ failure (i.e., oliguria, renal failure, or lactic acidosis). Renal replacement therapy was used according to previously published criteria.[21] Standard medical treatment for the suspected cause was introduced immediately after the initial clinical evaluation (e.g., antimicrobials, anticonvulsants, or antidotes).

### Etiological diagnosis

Computed tomography (CT) or magnetic resonance imaging (MRI) was obtained routinely, and any abnormal findings were described as focal and/or diffuse and as transient or permanent. Lumbar puncture and intermittent EEG monitoring were performed as needed. Cerebrospinal fluid (CSF) abnormalities were defined as a cell count >5/μL, protein >45 mg/dL, glucose <0.5 blood glucose, organisms, and/or atypical cells.[22] Electrical seizure activity was defined as continuous or recurrent, rhythmic, focal or generalized spikes; sharp waves; spike waves; or rhythmic waves changing in amplitude, frequency, and/or spatial distribution.[23] Laboratory tests were obtained routinely to look for metabolic abnormalities associated with neurological manifestations such as disturbances in serum sodium, calcium, urea, or glucose. Blood cell counts and coagulation tests were done to assess the bleeding risk. Plasma anticonvulsant-drug assays and qualitative tests for toxic substances or medications associated with coma were performed as indicated by the clinical history. A neurosurgical biopsy was performed only when focal lesions visualized by cerebral imaging studies remained of unknown or doubtful nature despite extensive investigations. The bedside physician was asked whether the etiological investigations contributed directly to the final diagnosis.

In each patient, the etiologic diagnosis was made by consensus among the intensivists, hematologists, and consultant neurologists. CNS infiltration by the malignant cells was diagnosed in patients meeting at least two of the following criteria: positive autopsy or biopsy, typical neuroimaging findings, atypical cells identified by cytology and/or histopathology in marrow smears or bone biopsies, and/or neuroimaging and clinical response to specific chemotherapy. Drug-related neurological complications were defined as adverse neurological events occurring after exposure to a drug known to induce neurological toxicity. Cerebrovascular disease was diagnosed based on neuroimaging evidence of acute ischemic or hemorrhagic stroke, cerebral venous thrombosis, or subarachnoid hemorrhage.

Metabolic CNS injury was considered when neuroimaging and CSF studies were normal and laboratory tests showed metabolic disturbances. Finally, CNS infection was diagnosed in patients with clinical evidence of sepsis, positive serological and/or microbiological tests on CSF.

### Statistical analysis

Quantitative parameters are reported as median and interquartile range (IQR, 25th -75th percentile) and qualitative parameters as number and percentage. Categorical variables were compared using the χ^2^ test or Fisher’s exact test, as appropriate. Continuous variables were compared using the Mann-Whitney U test or Wilcoxon test, as appropriate.

The primary outcome was vital status at hospital discharge, which was known for all patients. Associations linking patient characteristics to hospital mortality were assessed using a logistic regression model. Factors included in the multivariate regression model were selected among variables yielding *P* values smaller than 0.05 and/or were clinically relevant by univariate analysis. A stepwise procedure based on the Akaike Information Criterion was used to build the final multivariate model. Log-linearity of continuous variables was checked. Goodness of fit was evaluated by applying the Le Cessie-Van Houweligen test to the final models. Odds ratios (OR) and their 95% confidence intervals (95%CI) were computed. The secondary objective was to identify associations between patient characteristics and 1-year mortality. A Cox proportional hazard model was built for each variable and the proportional hazards assumption was assessed using Schoenfeld residuals. A multivariate model was built with variables that yielded *P* values smaller than 0.05 by univariate analysis and/or were clinically relevant (Survival *R*-package). There were 67 observations deleted due to missingness. One-year adjusted survival curves for cox proportional hazards model are reported according to neurological failure occurrence (Survminer *R*-package). *P* values less than 0.05 were considered statistically significant. Analyses were done using R software version 3.1.2. (online at http://www.R-project.org).

## Results

### Study population

Fig 1 is the study flow chart. In the 1011 patients with hematological malignancies who required ICU admission, the median number of organ failures was 2 (IQR 1–2) and varied across underlying diseases (Table 1). Neurological failure was the fourth most common organ failure, with 226 (22.4%) patients at ICU admission. The main characteristics of these 226 patients are reported in Fig 1.

A history of cancer chemotherapy within 30 days before ICU admission was noted in 95 (42%) patients, and 15 (6.6%) patients had received radiotherapy.

### Neurological presentation at ICU admission

**Fig 2:**
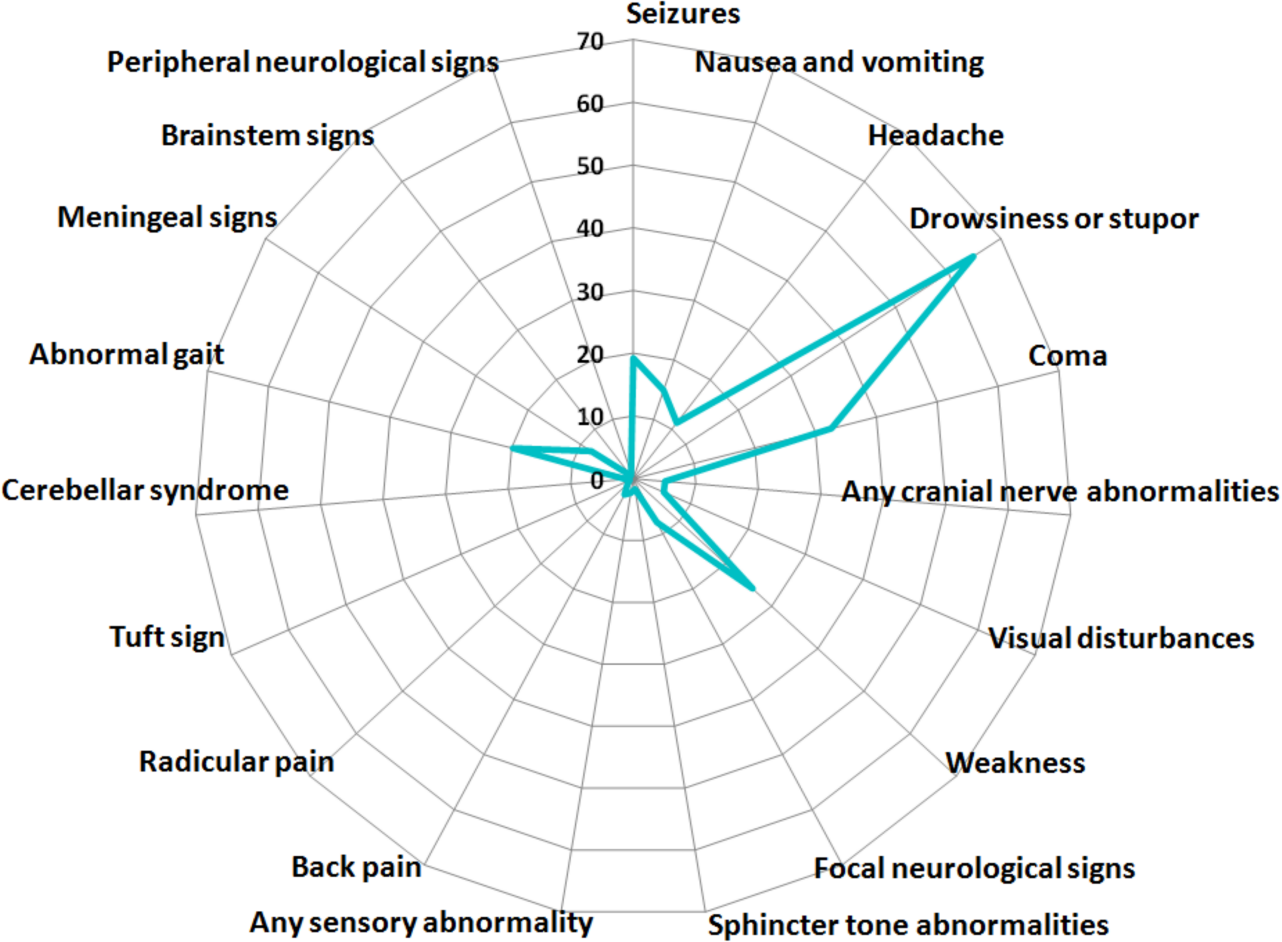
Neurological signs and symptoms in 226 patients with hematological malignancies and neurological failure requiring ICU admission.

Table 2 lists and Fig 2 illustrates the neurological features at ICU admission. Median GCS score was 12 (7–15). Neuroimaging, lumbar puncture, and electroencephalography were performed in 113 (50%), 73 (32%), and 63 (28%) patients, respectively, and were deemed directly contributive to the final diagnosis in 51 (45%), 18 (25%), and 43 (38%) patients, respectively. A neurosurgical biopsy was performed in a single patient and provided the etiological diagnosis. Neurological involvement was direct in 11 (5%) patients, due to indirect neurological factors in 114 (50%) patients (metabolic, n = 73; CNS infection, n = 12; vascular damage, n = 14, and iatrogenic, n = 15), due to indirect nonneurological factors in 77 (34%) patients (shock, n = 52; sepsis, n = 10; and cardiac arrest, n = 15), and due to unidentified factors in 24 (11%) patients. The anatomic sites of involvement were as follows: hemispheres, n = 200 (88%); meninges, n = 5 (2%); brainstem, n = 2; cerebellum and/or spinal cord, n = 1; and undetermined, n = 17 (8%).

### ICU management

The median SAPS II at ICU admission was 58 (47–70) and the median SOFA score on the day after ICU admission was 9 (5–13) indicating a total of 3 (2–3) organ failures. At ICU admission, endotracheal mechanical ventilation was required in 124 (54.9%) patients and noninvasive ventilation in 17 (7.5%) patients. Table 3 provides further data on ICU management.

### Outcomes after ICU admission

**Fig 3:**
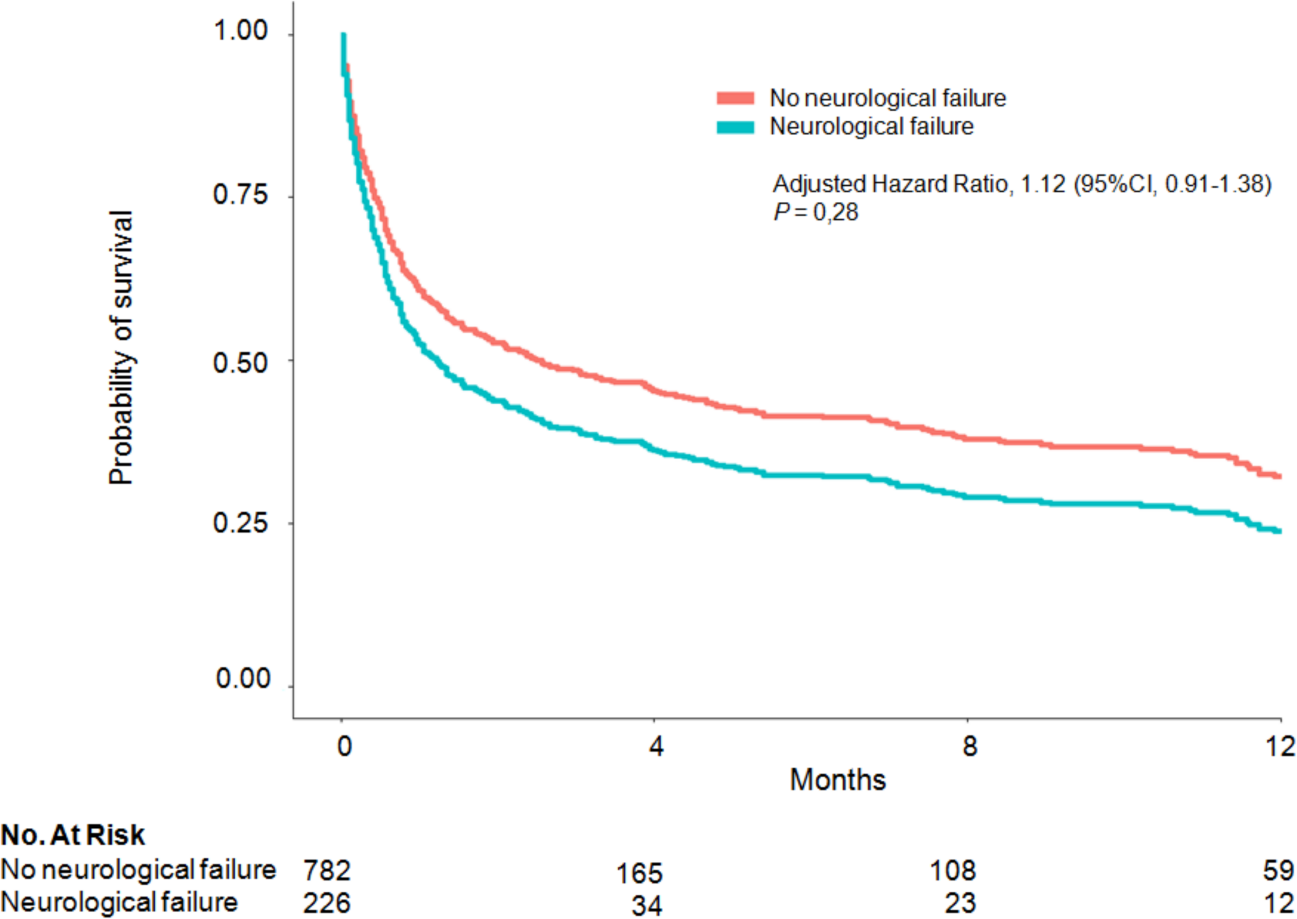
One-year adjusted survival curves for cox proportional hazards model with (blue line) and without (red line) neurological failure at ICU admission.

In the 226 patients with neurological failure, ICU and hospital stay lengths were 5 (2–12) and 30 (11–48) days, respectively. Hospital mortality was 49.6%. Mortality was 53.6% on day 30 after ICU admission and 58.4% after 1 year. Survival after 1 year was not significantly lower in the 226 patients with neurological failure than in the other 785 patients (aHR, 1.12; 95%CI, 0.91–1.38; *P* = 0.28) (S1 Table and Fig 3).

### Determinants of hospital mortality

By multivariate analysis, independent predictors of higher hospital mortality were poor performance status (OR, 3.99; 95%CI, 1.82–9.39; *P* = 0.0009), non-Hodgkin’s lymphoma (OR, 2.60; 95%CI, 1.35–5.15; *P* = 0.005), shock (OR, 1.95; 95%CI, 1.04–3.72; *P* = 0.04), and respiratory failure (OR, 2.18; 95%CI, 1.14–4.25; *P* = 0.02). Factors associated with lower hospital mortality were a higher GCS score on day 1 (OR, 0.88/point; 95%CI, 0.81–0.95; *P* = 0.0009) and autologous stem cell transplantation (OR, 0.25; 95%CI, 0.07–0.75; *P* = 0.02).

## Discussion

This multicenter cohort study is the largest to date providing prospective data on neurological complications in critically ill patients with hematological malignancies. Neurological failure was common, protean in its presentation, and related chiefly to indirect factors, both neurological and nonneurological. Mortality was high, with most deaths occurring within the first month. By multivariate logistic regression analysis, autologous stem cell transplantation was associated with higher short-term survival; factors associated with lower survival were type of hematological malignancy, poor performance status, hemodynamic and respiratory failures, and degree of consciousness impairment.

Studies have described the management and outcome of critically ill patients with hematological malignancies and multiple organ failures, which included neurological failure in 2.4% to 16.9% of cases. The study designs vary but are usually retrospective, the study periods differ, and neurological failure was often defined as coma, so that no data on the many other presentations are reported.[24–29] Of the few studies evaluating the neurological complications of malignancies, one was retrospective and confined to critically ill patients with any type of malignancy admitted over the 8-year period from 2000 to 2007[10] and another included only patients who had malignancies treated with hematopoietic transplantation.[30] Here, we sought to avoid previous methodological shortcomings by using a prospective multicenter design and a previously validated definition of neurological failure that better reflects the presentations seen in everyday practice.

Neurological failure occurred in 22.4% of all ICU patients with hematological malignancies. Non-Hodgkin’s lymphoma and acute myeloid leukemia were the most common diseases. Compared to our earlier study,[10] the proportion of newly diagnosed hematological malignancies was considerably higher (40% vs. 16%), whereas findings were similar for the time from malignancy diagnosis to neurological failure and for the proportions of patients in remission, having received chemotherapy within the last 4 weeks, having a history of hematopoietic transplantation, or having neutropenia at presentation.[10]

The full spectrum of neurological complications of hematological malignancies was represented. The high proportion of patients with seizures (19.2%) may be ascribable to the nature of the malignancies in our population. In earlier studies, seizures occurred in 5% of patients after hematopoietic transplantation,[30] 8% of patients with non-Hodgkin’s lymphoma,[31] and 20% of patients with acute myeloid leukemia.[32] Another factor may be that some of our patients had postanoxic status epilepticus after cardiac arrest.[10, 33] The cause of neurological failure was identified in the vast majority of our patients. Depending on the nature of the investigation, a direct contribution to the diagnosis was reported in 25% to 100% of cases. The previously used classification into direct and indirect causes of CNS involvement proved inadequate in describing the full spectrum of cases.[11, 13] Thus, nonneurological causes such as cardiac arrest,[2] severe sepsis, and shock were involved in about one-third of cases. The proportions of patients with other causes were in keeping with our earlier report.[10]

Most patients had multiple organ failures at ICU admission. Mechanical ventilation was the most often used life-supporting intervention, probably because many patients were comatose. Catecholamines and dialysis were also often required at ICU admission. This high level of life support is consistent with the changes in management strategies that have accompanied recent advances in the management of malignancies.[3, 4, 34, 35] ICU admission criteria have been developed jointly by hematologists and intensivists, in particular to ensure early admission of patients with incipient organ failure, and full-code treatment is then given routinely until reevaluation of the prognosis after a few days.[8, 9]

Despite the extensive etiological investigations and high level of organ support, mortality was high, with nearly half the patients dying in the hospital. Among 57 non-ICU patients with neurological complications after hematopoietic transplantation, 21 (37%) died.[30] In a single-center retrospective study of 124 critically ill patients with hematological malignancies, mortality in the subgroup admitted for neurological impairment was 33%.[24] Another retrospective study, in 100 patients admitted to the ICU for neurological failure, found an inhospital mortality rate of 45%.[10] After hematopoietic transplantation, the occurrence of neurological complications was associated with mortality.[30]

We identified several factors independently associated with hospital mortality. Autologous stem cell transplant recipients are generally at high risk for death, and their better survival in our study reflects good selection of patients likely to benefit from ICU admission. However, despite the use of effective ICU-admission criteria, poor performance status, hemodynamic and respiratory failure in addition to neurological failure, and greater coma severity were associated with a higher risk of death. Finally, hospital mortality was higher in patients with non-Hodgkin’s lymphoma, in keeping with the nearly exclusive occurrence of CNS involvement in the aggressive forms of this disease, which are also associated with high mortality.[36],[37, 38]

Our study has several limitations. Consultants were available around the clock in the participating centers, and our findings may not apply to hospitals where this is not the case. Although great care was taken to identify, report, and explain neurological involvement in our prospective cohort of 1011 ICU patients with hematological malignancies, this study is a post-hoc analysis. However, the standardized form used to prospectively collect data on neurological involvement, combined with the participation of 17 university or university-affiliated centers, provides a comprehensive picture of the management and prognosis of the population of interest. The etiology was identified and treated in the vast majority of patients. Finally, the ICU-admission criteria may have varied across study centers. However, the proportions of patients who were refused ICU admission did not differ significantly across centers, and refusal was not associated with hospital mortality.

In conclusion, in patients with hematological malignancies who require ICU admission, neurological failure is common and associated with a high risk of death. Factors independently associated with death are type of malignancy, poor performance status, hemodynamic and respiratory failures, and severe consciousness impairment. Knowledge of these risk factors may help to improve management strategies.

## Supporting information

S1 Table Variables associated with 1-year mortality in 1011 patients with hematological malignancies admitted to the intensive care unit. (DOCX)

## Abbreviations

ICU: intensive care unit
CNS: central nervous system
GCS: Glasgow Coma Scale
SAPS-II: Simplified Acute Physiology Score II
SOFA: Sepsis-Related Organ Failure Assessment
CT: Computed tomography
CSF: Cerebrospinal fluid
