# Supplementary material for: Neurological failure in ICU patients with hematological malignancies: A prospective cohort study
Source: PLoS One. 2017 Jun 9;12(6):e0178824. doi: 10.1371/journal.pone.0178824 (PMC5466302; doi:10.1371/journal.pone.0178824)
Supplement: S1 Table — (DOCX) [file pone.0178824.s001.docx]

**S1 Table: Variables associated with 1-year mortality in 1011 patients with hematological malignancies admitted to the intensive care unit**

|  | Univariate analysis | | Multivariable analysis | |
| --- | --- | --- | --- | --- |
|  | Crude HR (95%CI) | *P* value | Adjusted HR (95%CI) | *P* value |
| **Patient characteristics** |  |  |  |  |
| Age (years), median (IQR) | 1.01 (1.00-1.02) | 0.0002 | 1.01 (1.00-1.02) | <0.00001 |
| Male gender | 1.14 (0.95-1.37) | 0.17 |  |  |
| Poor performance status^a^ | 1.88 (1.53-2.29) | <0.00001 | 1.66 (1.35-2.05) | <0.00001 |
| **Characteristics of the underlying malignancy** |  |  |  |  |
| Classification |  |  |  |  |
| Non-Hodgkin’s lymphoma | 1.02 (0.85-1.24) | 0.81 |  |  |
| Acute myeloid leukemia | 1.11 (0.91-1.35) | 0.31 |  |  |
| Myeloma | 0.77 (0.57-1.03) | 0.08 | 0.71 (0.52-0.96) | 0.03 |
| Acute lymphocytic leukemia | 0.88 (0.63-1.24) | 0.46 |  |  |
| Chronic lymphocytic leukemia | 1.07 (0.77-1.45) | 0.69 |  |  |
| Myelodysplastic syndrome | 1.05 (0.69-1.60) | 0.80 |  |  |
| Chronic myeloid leukemia | 0.53 (0.22-1.29) | 0.16 |  |  |
| Hodgkin’s disease | 0.86 (0.46-1.60) | 0.62 |  |  |
| Other | 1.42 (0.95-2.11) | 0.08 | 1.31 (0.86-2.00) | 0.21 |
| Newly diagnosed at hospital admission | 0.88 (0.73-1.06) | 0.19 |  |  |
| Allogeneic stem cell transplantation | 1.40 (1.11-1.76) | 0.004 | 1.96 (1.49-2.57) | <0.00001 |
| Autologous stem cell transplantation | 0.93 (0.71-1.22) | 0.60 |  |  |
| Remission (complete or partial) | 0.80 (0.64-1.00) | 0.05 | 0.66 (0.52-0.85) | 0.03 |
| **Circumstances and severity scores at ICU admission** |  |  |  |  |
| SAPS II^b^, median (IQR) at ICU admission | 1.04 (1.03-1.04) | <0.00001 |  |  |
| SOFA score^b^, median (IQR) at ICU admission | 1.15 (1.13-1.18) | <0.00001 |  |  |
| Neurological failure at ICU admission | 1.31 (1.07-1.60) | 0.009 | 1.12 (0.91-1.38) | 0.28 |
| **Treatment in the ICU** |  |  |  |  |
| Catecholamine support during ICU stay | 2.35 (1.94-2.85) | <0.00001 | 1.52 (1.19-1.94) | 0.0009 |
| Noninvasive ventilation during ICU stay | 1.23 (1.02-1.49) | 0.03 |  |  |
| Mechanical ventilation during ICU stay | 2.46 (2.06-3.02) | <0.00001 | 1.78 (1.40-2.26) | <0.00001 |
| Dialysis during ICU stay | 1.62-1.35-1.95) | <0.00001 | 1.43 (1.17-1.74) | 0.0004 |
| Emergent anticancer chemotherapy during ICU stay | 0.73 (0.48-1.09) | 0.13 |  |  |

HR, Hazard Ratio; 95%CI, 95% confidence interval;; ICU, intensive care unit; SAPS II, Simplified Acute Physiology Score; SOFA score, Sequential Organ Failure Assessment score

^a^bedridden/completely disabled

^b^Higher scores indicate greater severity.
